# Preventive Effects of a Human Hematopoietic Mesenchymal Stem Cell (hHMSC) Therapy in Ovalbumin-Induced Food Allergy

**DOI:** 10.3390/biomedicines10020511

**Published:** 2022-02-21

**Authors:** Dong-Geon Lee, Yu-Jin Lee, Song-Hee Park, Hye-Ree Park, Hoon Kang, Jung-Eun Kim

**Affiliations:** Department of Dermatology, Eunpyeong St. Mary’s Hospital, College of Medicine, The Catholic University of Korea, Seoul 03312, Korea; dlcjdgnl@naver.com (D.-G.L.); cindyeujine1@naver.com (Y.-J.L.); saccharide@hanmail.net (S.-H.P.); 5953hari@naver.com (H.-R.P.); johnkang@catholic.ac.kr (H.K.)

**Keywords:** human mesenchymal stem cell, food allergy, prevention

## Abstract

No effective therapeutic strategies have been developed against food allergies. Immunomodulation during early infant period could prevent the development of food allergies. We investigated the preventive effects of human hematopoietic mesenchymal stem cells (hHMSCs) in mice with ovalbumin (OVA)-induced food allergy. BALB/c mice with OVA-induced food allergy were divided into 3 groups, and each group was treated with hHMSCs or hHMSC culture medium (hHMSC-CM) or saline. Ear thickness, allergy score, rectal temperature, and diarrhea occurrence were checked. Total IgE, OVA-specific IgE, and mucosal mast cell protease-1 (mMCP-1) were measured by ELISA. Other allergic parameters were analyzed using histology specimens, RT-PCR, and flow cytometry. Treatment with hHMSCs or hHMSC-CM significantly suppressed the frequency of anaphylactic response and rectal temperature decline, reduced diarrhea, total IgE, OVA-specific IgE, and mMCP-1. While the treatment decreased the level of Th2 cytokines, it enhanced IL-10 and TGF-β1 mRNA. Exposure to hHMSC or hHMSC-CM did not generate regulatory T cells, but reduced mast cells. The immunomodulatory effect on the Th2 cytokines was greater in hHMSC-CM than in hHMSCs. hHMSC treatment may be a promising preventive intervention against food allergy. Further studies are needed to elucidate the key substances released from hHMSC to induce immune tolerance.

## 1. Introduction

Human hematopoietic mesenchymal stem cells (hHMSCs) are a type of mesenchymal stem cells (MSCs), which can differentiate into multi-lineage cells. MSCs are thought to have anti-inflammatory and immunosuppressive effects, inhibiting proliferation and differentiation of T cells and B cells by secreting interleukin (IL)-10, tumor growth factor-beta (TGF-β), and prostaglandin E2 (PGE2) [1,2]. Moreover, systemic administration of MSCs suppresses T cell-mediated responsiveness, promotes regulatory cell differentiation [3,4], and modifies the abundance and composition of the gut flora [5]. Several studies have reported that MSCs can alleviate signs of allergic diseases such as atopic dermatitis (AD) [6] and asthma [7], but few studies to date have reported the effects of hHMSC on food allergy.

The clinical manifestations of food allergy vary. Most common clinical symptoms and signs are pruritus and acute urticaria, however sometimes anaphylaxis may lead to lethal results. The etiological food allergens are different depending on the patients’ age. Whereas eggs, cow milk, and flour are common food allergens for infants, nuts and crustaceans are the main causes for adults. Food allergy to eggs, cow milk, and flour can disappear as a child grows, however, food allergy to nuts or crustaceans usually persist throughout a lifetime [8]. The pathophysiology of food allergy is complex. Some AD infants develop food allergy during childhood through skin sensitization, so called allergic march. Certain patients with food allergy only have gastrointestinal symptoms and never develop other allergic comorbidities such as AD or asthma [9]. Food allergies can be classified into immunoglobulin (Ig)E-mediated and non-IgE-mediated allergy. In the IgE-mediated food allergy, the allergen induces T helper (Th)2 response and IgE binding to Fcε receptors on the mast cells, releasing histamine [10]. Pathophysiology of non-IgE-mediated food allergy is poorly understood, though IL-2, IL-4, IL-5, IL-13, IL-17, tumor necrosis factor (TNF)-α, and TGF-β are thought to be associated [11]. In our study, we speculated that ovalbumin (OVA) challenge induces IgE-mediated food allergy. IgE-mediated food allergy can be easily recognized from patients and physicians because the symptoms immediately develop after exposure to food allergen. However, non-IgE-mediated food allergy is hard to diagnose even for physicians, and repeated clinical history is crucial to precise diagnosis. 

Food allergy is difficult to manage. Many patients with food allergy live with risk of emergency and rely on self-epinephrine injection during anaphylaxis episodes. Despite the fact that IgE contributes as a key player in the pathogenesis of food allergy, omalizumab, a monoclonal antibody for IgE Fc region, which is indicated for asthma and allergic rhinitis with nasal polyps, it is not obviously effective in the treatment of food allergy [12]. Although the prevalence is gradually increasing, no effective strategies have been developed except avoidance of the offending food. Probiotic modulation of the gut microbiota composition during early infancy has been used to prevent the development of allergic diseases [13]. Similarly, immunomodulation during early infant period prevents the development of food allergies [14]. As food allergy is known to be caused by oral intolerance and a defective host barrier [15], the immunosuppressive effects of hHMSCs improve food allergy symptoms, suggesting a potential method for treating allergic diseases.

Although MSCs that originated from bone marrow are the most widely studied in allergic diseases, MSCs can be obtained from many different tissues such as umbilical cord and adipose tissue. Changes in immune cells and cytokines after MSC treatment have been described in previous studies involving MSCs in allergic diseases. In the asthmatic mouse model, intravenous injection of bone marrow-derived MSCs decreased the levels of total IgE, IL-5, and IL-13 [7], human-induced pluripotent stem cells decreased the levels of OVA-specific IgE, IgG, IL-4, IL-5, and IL-13 [16], and placenta-derived MSCs increased IL-10 and regulatory T (Treg) cells [17] in the serum. Adipose tissue-derived MSCs decreased IL-4, IL-5, and IL-13 [18] in the bronchoalveolar lavage fluid (BALF), increased IL-10, IFN-γ, and TGF-β in the lung [19], BALF [18], lung lymph node [18], and Treg cells in the spleen [19], and lung lymph nodes [18]. Intratracheal administration of bone marrow-derived MSCs decreased OVA-specific IgG in the serum, and decreased IL-4 and increase IL-12 in both serum and BALF [20]. In a mouse model of allergic rhinitis, nasal mucosa-derived MSCs enhanced the Th1 immune response by upregulating IFN-γ levels, while they inhibited the Th2 immune response by downregulating IL-4, IL-5, and IL-10 expression [21]. In a mouse model of allergic conjunctivitis, topical administration of TNF-α-stimulated bone marrow-derived MSC culture medium decreased mast cell activity, IgE, and histamine levels [22]. In an AD mouse model, intravenous administration of MSCs reduced IgE levels in the serum, inhibited B cell differentiation, T cell activities, and cytokine production [6]. In most previous studies, MSCs were administered as an intravenous injection. An intravenous or subcutaneous administration of MSCs appear to act systemically and locally, respectively, and are equally effective, although intravenous administration was more effective in one study involving AD mouse model [23].

Cytokine secretion of MSCs seems to be varied among origin [24,25], but there have been no studies directly comparing the effects of MSCs on food allergy by origin. In our study, MSCs from bone marrow were used, which have been most commonly used in previous studies on allergic diseases, as well as which can be easily obtained in a less invasive manner.

We investigated the preventive effects of intravenous hHMSC therapy in mice with OVA-induced food allergy.

## 2. Materials and Methods

### 2.1. Sensitization and Challenge with OVA in a Mouse Model

The animal experiments were performed according to the Guidelines for the Care and Use of Laboratory Animals and were approved by the Institutional Animal Care and Use Committee of Central Bio (19311ET1). A total of 42 male BALB/c mice aged 6 weeks were obtained from Samtacobio Co., Osan, Korea. 

**Figure 1:**
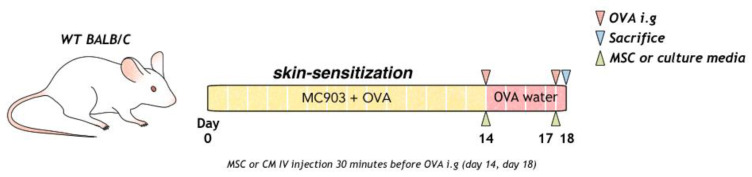
Process of allergic sensitization to food in mice with OVA, MSC, or MSC CM is shown. MSC, mesenchymal stem cell; MSC CM, mesenchymal stem cell culture medium; OVA, Ovalbumin.

To induce food allergy, topical Daivobet (Calcipotriol 0.005%, LEO Pharma A/S, Copenhagen, Denmark) 20 µg and ovalbumin (OVA) (#A5503, Sigma Aldrich, Boston, MA, USA) 100 µg/head/PBS were applied at 10-min intervals to mouse on the dorsal skin every day for 2 weeks. Mice were intra-gastrically challenged with 2 g ovalbumin (50 mg/200 µL) on days 14 and 18, as previously described. OVA (1.5 g/L) was provided in the water between days 14 and 18 (Figure 1).

### 2.2. Intravenous Human MSC or MSC-CM Administration into BALB/c Mice

We obtained human bone marrow-derived mesenchymal stem cells (Catholic MASTER Cells from the Catholic Institute of Cell Therapy (CIC, Seoul, Korea)). 

Cells were derived from human bone marrow donated by healthy donors after informed consent. The use of stem cells in the experiments was approved by the Institutional Review Board (PC21TESI0013). The hHMSCs in passages 5 were used for the experiments. The culture media was Dulbecco’s Modified Eagle Medium (DMEM high glucose, Gibco BRL, Life Technology, Karlsruhe, Germany) containing 10% fetal bovine serum (FBS; Gibco BRL, Life Technology, Karlsruhe, Germany) and 1% penicillin/streptomycin (Gibco, BRL).

Culture medium was concentrated with Amicon^®^ Pro Purification System (EMD Millipore Corporation, Billerica, MA, USA) using 3000-Da pore size membrane and desalted with an HiTRAP desaltin kit (GE Healthcare, Uppsala, Sweden). Culture medium was stored at −70 °C before usage. Mice were divided into four groups. Each group was treated with hHMSCs (n = 12) or hHMSC-CM (n = 12), or saline (n = 12) in the OVA-induced allergic mice and compared with normal BALB/c mice (n = 6). Mice in different groups were intravenously treated with saline, 1 mL/kg (20 µL per mouse) of the hHMSC-CM concentrates, or the hMSCs (1.0 × 10^5^ cells) were intravenously administered through tail vein 30 min before the intragastric challenge of OVA on days 14 and 18.

### 2.3. Clinical Symptoms

Allergy was scored immediately after the last challenge of OVA using a 7-point Likert scale as follows [26]: 0, no symptoms; 1, less than 4 episodes of scratching and rubbing around nose and head; 2, 4–10 episodes of scratching and rubbing around nose and head; 3, more than 10 episodes of scratching and rubbing around nose and head; 4, hunching and piloerection; 5, immobility (unresponsive to nonharmful, tactile disturbance); and 6, death).

Diarrhea, and anaphylactic responses was measured with 4-point Likert scales [27]: 0, normal stools; 1, a few wet and unformed stools; 2, a number of wet and unformed stools with moderate perianal staining of the coat; and 3, severe and watery stools with severe perianal staining of the coat. Anaphylactic responses were measured with 5-point Likert scales: 0, no symptoms; 1, reduced activity, trembling of limbs; 2, loss of consciousness, no activity upon prodding; 3, convulsions; and 4, death. The scores were assessed 20 min after the last injection of OVA as previously described [27]. Rectal body temperature was measured before the last injection of OVA and at the end of observation of clinical symptoms.

### 2.4. Measurements of Total IgE, OVA-Specific IgE, IgG1, and mMCP-1 Levels

After measuring body temperature, serum was harvested from the surviving mice and stored at −70 °C before usage. Concentrations of total IgE (AKRIE-010, FUJIFILM Wako Shibayagi Corporation, Shibukawa, Gunma, Japan), OVA-specific IgE (AKRIE-030, FUJIFILM Wako Shibayagi Corporation, Shibukawa, Gunma, Japan), OVA-specific IgG1 (Cayman, Item. No. 500830), and mucosal mast cell protease-1 (mMCP-1, # 88-7503, Thermo Fisher Scientific, Waltham, MA, USA) in the serum were measured using an ELISA kit. Euthanasia was performed by an experienced technician by cervical dislocation at day 18 after checking clinical symptoms and obtaining serum and tissue.

### 2.5. Histopathological Analysis

Formalin-fixed, paraffin-embedded tissue sections were subjected to hematoxylin-eosin staining for microscopic examination of ear skin, small intestine (SI), and spleen. Toluidine blue staining of mast cells with ear skin and transverse colon were used to evaluate edema, the thickness of ear skin, and colon. The thickness of colon was measured from the bottom of intestinal villi to submucosa of jejunum or ileum. The villus/crypt ratio was calculated in each group as previously described [28].

Mast cell count (%) was quantified using a digital imaging software program, ImageJ (Version 1.8.0, National Institutes of Health, Bethesda, MD, USA; available at http://rsb.info.nih.gov (accessed on 17 May 2021).

### 2.6. Measurements of Cytokines and Chemokines by qPCR

Total RNA was extracted from the homogenized tissues of ear skin and SI using AccuPrep^®^ Universal RNA Extraction Kit (Cat.K-3140, Bioneer, Daejeon, Korea), and cDNA was synthesized with AccuPower^®^ 2× GreenStar Master Mix (Cat.K-6253, Bioneer, Daejeon, Korea) according to the manufacturer’s instructions. Concentrations of IL-4, IL-5, IL-13, IL-10, IL-23, IL-31, IL-12, IFN-γ, and TGF-β1 in the tissues of ears and SI were determined using AccuPower^®^ qPCR Array (Bioneer, Daejeon, Korea). Detailed gene information is presented in Supplementary Table S1.

### 2.7. Flow Cytometry

Flow cytometry was performed to determine the changes in the number of CD4+ Foxp3+ regulatory T cells and IgE+ c-kit+ mast cells in the ear skin, SI, and mesenteric lymph nodes in response to MSCTs. Treg cells were defined as CD4+/FoxP3+ high cells, as previously described [29]. Suspended T cells were stained with a mouse regulatory T cell staining kit #3 (Cat. No. 88-8115-40, eBioscience™, Shanghai, China) and the suspended mast cells were stained with a CD117 (c-Kit) antibody (Cat. No.12-1171-82, eBioscience™, Shanghai, China) and FceR1 alpha monoclonal antibody (MAR-1) (Cat. No. 12-5898-82, eBioscience™, Shanghai, China). Stained cells were resuspended and fixed with 1% paraformaldehyde 500 μL. All data were collected on a FACSCalibur (Becton Dickinson, Franklin Lakes, NJ, USA) and analyzed with CellQuest software (Version 5.2, BD Biosciences, San Jose, CA, USA).

### 2.8. Statistical Analysis

All values are expressed as mean ± standard error of the mean. Statistical comparisons between groups were performed. For parametric variables, the Scheffe test was used for equal variance, and the Dunn’s test and Tukey’s multiple comparison test were used for heteroscedasticity. For nonparametric statistics, the Kruskal–Wallis test was used, followed by the Bonferroni post-hoc test.

## 3. Results

### 3.1. Allergy Scoring, Diarrhea, Anaphylactic Responses, and Body Temperature Changes

We investigated the effect of hHMSC and hHMSC culture medium (hHMSC-CM) on alleviation of clinical allergic response. Elevated allergy score in the OVA-challenged mice (OVA mice) decreased with hHMSCs (hHMSC mice) or hHMSC-CM (hHMSC-CM mice). Likewise, diarrhea and anaphylactic responses in the OVA mice were improved in both hHMSC mice and hHMSC-CM mice. Effects of intervention on both allergy scoring and diarrhea were higher in the hHMSC-CM mice. Decrease in body temperature with OVA challenge was similarly relieved in mice exposed to hHMSC and hHMSC-CM (Table 1).

### 3.2. Total IgE, OVA-Specific IgE, OVA-Specific IgG, and mMCP-1

**Figure 2:**
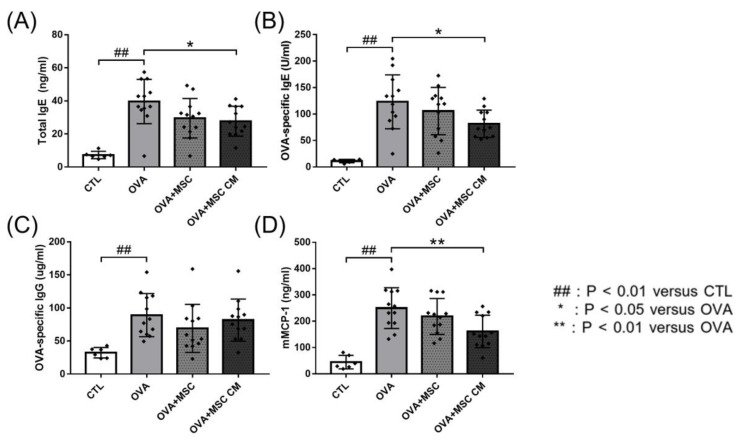
OVA challenge elevated immunoglobulin and mMCP-1 levels and MSC or MSC CM reversed the change. Concentrations of (**A**) total IgE, (**B**) OVA-specific IgE, (**C**) OVA-specific IgG, and (**D**) mMCP-1 in the serum measured by ELISA are shown. Each value indicates mean ± standard error of the mean. ## *p* < 0.01 versus CTL, * *p* < 0.05 versus OVA, ** *p* < 0.01 versus OVA. CTL, control; Ig, immunoglobulin; mMCP, mucosal mast cell protease; MSC, mesenchymal stem cell; MSC CM, mesenchymal stem cell culture medium; OVA, Ovalbumin.

Next, we investigated the effects of hHMSC and hHMSC-CM on total IgE, OVA-specific IgE, OVA-specific IgG, and mMCP-1 levels. In the OVA mice, total IgE, OVA-specific IgE, OVA-specific IgG, and mMCP-1 levels were all elevated. Addition of hHMSCs or hHMSC-CM decreased the IgE, IgG, and mMCP-1 levels, whereas hHMSCs showed no significant difference in all parameters, and hHMSC-CM induced a significant decrease in total IgE, OVA-specific IgE, and mMCP-1 levels (Figure 2).

### 3.3. mRNA Expressions of Genes Related to Cytokines in the Ear Skin and Small Intestine Tissue

**Figure 3:**
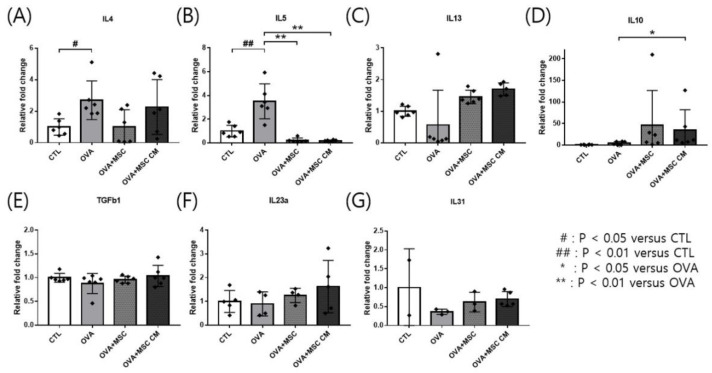
OVA challenge upregulated inflammatory cytokines and IL-10 transcription and MSC or MSC CM reversed the change in the ear skin. (**A**) IL-4, (**B**) IL-5, (**C**) IL-13, (**D**) IL-10, (**E**) TGF-β1, (**F**) IL-23α, and (**G**) IL-31 mRNA level changes in the ear skin measured by qPCR are shown. Each value indicates mean ± standard error of the mean. # *p* < 0.05 versus CTL, ## *p* < 0.01 versus CTL, * *p* < 0.05 versus OVA, ** *p* < 0.01 versus OVA. CTL, control; IL, interleukin; MSC, mesenchymal stem cell; MSC CM, mesenchymal stem cell culture medium; OVA, Ovalbumin; qPCR, quantitative polymerase chain reaction; TGF, transforming growth factor.

Transcription of cytokines was investigated in the ear skin and SI. IL-4 and IL-5 mRNA levels were upregulated in the ear skin of OVA mice. Addition of either hHMSCs or hHMSC-CM reversed the upregulation of IL-4 and IL-5, while no significant differences in IL-4 were detected Moreover, IL-10 was highly upregulated in mice exposed to hHMSCs and hHMSC-CM. The levels of IL-13, TGF-β1, IL-23α, and IL-31 mRNA in the ear skin showed no significant differences (Figure 3).

**Figure 4:**
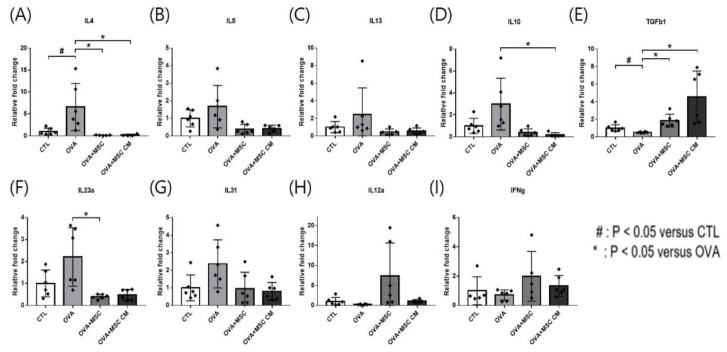
OVA challenge upregulated inflammatory cytokines and IL-10 transcription, downregulated TGF- β1 and MSC or MSC CM reversed the change in the SI. (**A**) IL-4, (**B**) IL-5, (**C**) IL-13, (**D**) IL-10, (**E**) TGF-β1, (**F**) IL-23α, (**G**) IL-31, (**H**) IL-12α, and (**I**) IFN-γ mRNA level changes in the SI measured by qPCR are shown. Each value indicates mean ± standard error of the mean. # *p* < 0.05 versus CTL, * *p* < 0.05 versus OVA, CTL, control; IL, interleukin; MSC, mesenchymal stem cell; MSC CM, mesenchymal stem cell culture medium; OVA, Ovalbumin; qPCR, quantitative polymerase chain reaction; SI, small intestine; TGF, transforming growth factor.

In the SI tissue, upregulated IL-4, IL-5, IL-13, IL-23α, and IL-31 in the OVA mice were downregulated in mice exposed to hHMSC and hHMSC-CM, though only changes in IL-4 and IL-23α were statistically significant. Elevated IL-10 transcription in OVA mice was significantly downregulated in the mice treated with hHMSC and hHMSC-CM, whereas the transcription of TGF-β1 was decreased in the OVA mice, and upregulated in mice treated with both hHMSC and hHMSC-CM. Downregulation of IL-12a and IFN-γ in the OVA mice was reverted in mice exposed to hHMSC and hHMSC-CM, although not statistically significantly so (Figure 4).

### 3.4. IgE+ c-kit+ Mast Cell Populations in the Ear Skin, Mesenteric Lymph Node, and SI Tissue

**Figure 5:**
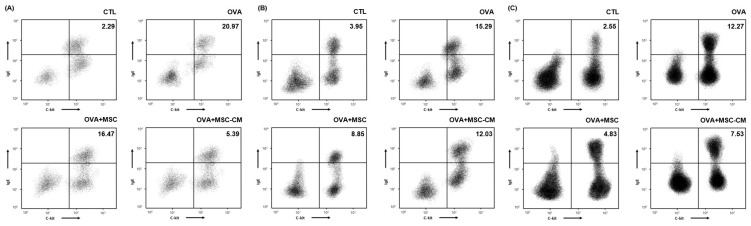
OVA challenge increased IgE+ c-kit+ mast cells and MSC or MSC CM reversed the change in the ear skin, mLN, and SI tissue. IgE+ c-kit+ mast cells in the (**A**) ear skin, (**B**) mLN, and (**C**) SI tissue measured by flow cytometry are shown. Mean percentage (mast cell counts/total cell counts) is shown in the right upper side. Ig, immunoglobulin; mLN, mesenteric lymph node; MSC, mesenchymal stem cell; MSC CM, mesenchymal stem cell culture medium; OVA, Ovalbumin; SI, small intestine.

The number of IgE+ c-kit+ mast cells substantially increased in the ear skin, mesenteric lymph node (mLN), and SI tissue in the OVA mice. Both hHMSC and hHMSC-CM decreased the mast cell population ratio in the ear skin, mLN, and SI tissue. The hHMSC-CM had a greater effect on the ear skin, and hHMSC had a greater effect on the mLN and SI tissues (Figure 5).

**Figure 6:**
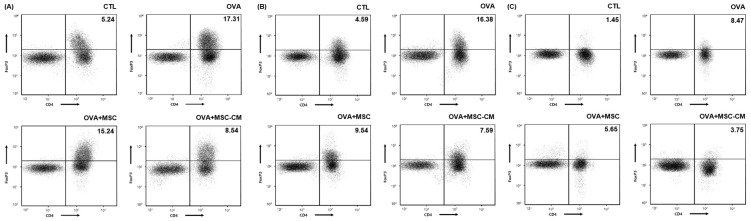
OVA challenge increased CD4+ Foxp3+ T cells and MSC or MSC CM reversed the change in the ear skin, mLN, and SI tissue. CD4+ Foxp3+ T cells in the (**A**) ear skin, (**B**) mLN, and (**C**) SI tissue measured by flow cytometry are shown. Mean percentage (CD4+ Foxp3+ T cell counts/total cell counts) is shown in the right upper side. CD, cluster of differentiation; Fox, forkhead box; mLN, mesenteric lymph node; MSC, mesenchymal stem cell; MSC CM, mesenchymal stem cell culture medium; OVA, Ovalbumin; SI, small intestine.

The levels of CD4+ Foxp3+ T cells also substantially increased in the ear skin, mLN, and SI tissue in the OVA mice. Both hHMSC and hHMSC-CM decreased the CD4+ Foxp3+ T cell populations in the ear skin, mLN, and SI tissue. The hHMSC-CM had a greater effect than hHMSC on ear skin, mLN, and SI tissues (Figure 6).

### 3.5. Histologic Changes in the Ear Skin, SI, and Spleen

**Figure 7:**
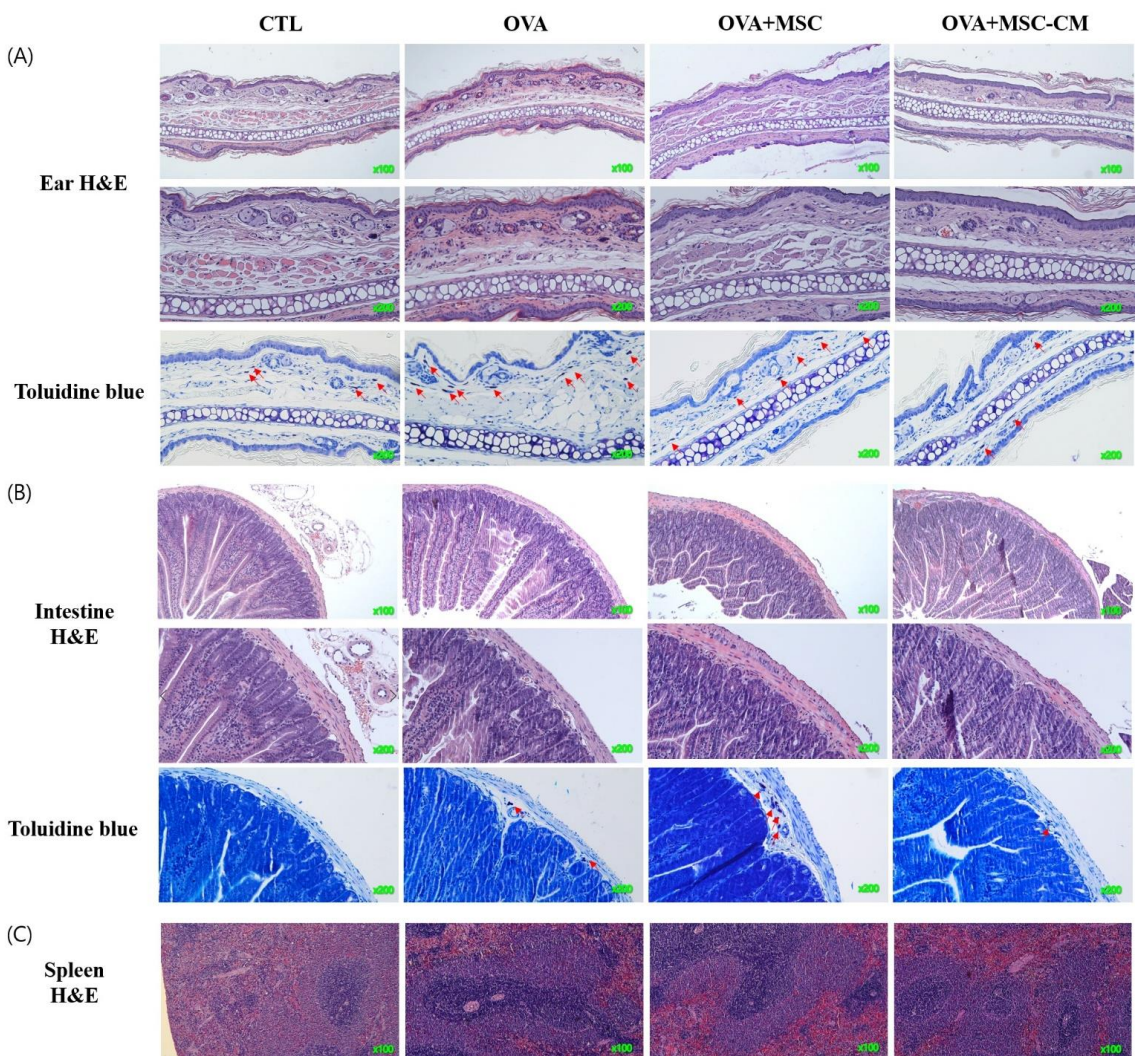
OVA challenge induced inflammation and mast cell infiltration in the tissue and MSC or MSC CM attenuated the change in the ear skin, SI, and spleen. Histological changes of (**A**) ear skin, (**B**) SI, or (**C**) spleen are shown. Sections are stained with H&E (hematoxylin and eosin, ear skin ×100, ×200, SI, ×100, ×200, spleen ×100) or Toluidine blue (×200). Mast cells are indicated by red arrows. H&E, hematoxylin, and eosin; MSC, mesenchymal stem cell; MSC CM, mesenchymal stem cell culture medium; OVA, Ovalbumin; SI, small intestine.

**Figure 8:**
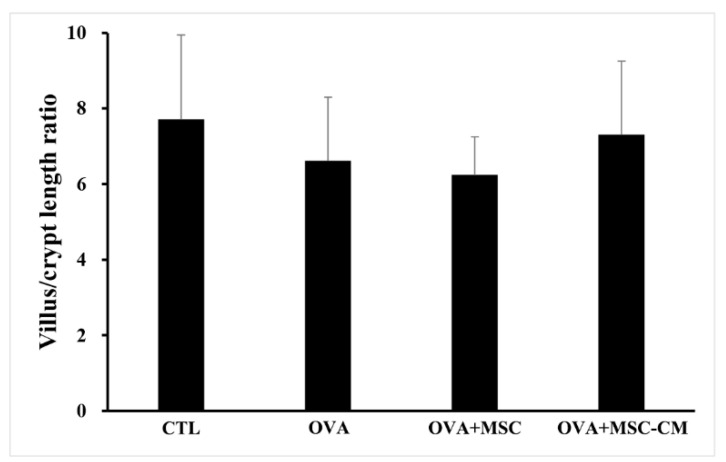
No significant changes of villus/crypt length ratio were observed in the colon with OVA, MSC, or MSC CM. Villus/crypt length ratio of colon is shown. Each value indicates mean ± standard error of the mean. MSC, mesenchymal stem cell; MSC CM, mesenchymal stem cell culture medium; OVA, Ovalbumin.

Inflammatory histologic changes, increase in mast cells, and thickening of the tissue were observed in the ear skin and SI of the OVA mice. Compared with OVA mice, the mice exposed to hHMSC and hHMSC-CM showed reduced levels of inflammatory cell infiltration along with decreased mast cell counts and tissue edema. Similarly, in the spleen, the increased weight and inflammatory cells in the OVA mice were reduced in the hHMSC mice and hHMSC-CM mice. Overall, histologic inflammation was reduced in hHMSC-CM more than in hHMSCs (Figure 7). No significant changes in villus/crypt length ratio of colon were detected in the OVA mice, hHMSC mice, or hHMSC-CM mice (Figure 8). Skin thickness, mast cell counts of ear skin and SI, and spleen weight of each group are shown in Supplementary Table S2.

## 4. Discussion

In our study, hHMSC or hHMSC-CM treatment significantly suppressed the frequency of oral OVA challenge-induced anaphylactic response and decline in rectal temperature. In the serum, elevated levels of total IgE, OVA-specific IgE, and mMCP-1 in the OVA-mice showed a significant decrease in mice treated with hHMSC-CM. In the ear skin, transcription of IL-5 increased, and IL-10 increased in mice exposed to hHMSC and hHMSC-CM. In the SI tissue, the upregulated IL-4 in the OVA mice was downregulated in mice exposed to hHMSC and hHMSC-CM, and IL-23α upregulation in the OVA mice was downregulated in the hHMSC mice. In the ear skin, IL-10 was enhanced by hHMSC or hHMSC-CM. In the SI tissue, IL-10 was induced by OVA and downregulated by hHMSC or hHMSC-CM. TGF-β1 was increased by hHMSC or hHMSC-CM. The number of IgE+ c-kit+ mast cells and CD4+ Foxp3+ T cells substantially increased in the ear skin, mLN, and SI tissues in the OVA mice, and decreased in the hHMSC and hHMSC-CM mice. In the ear skin and SI of mice treated with hHMSC and hHMSC-CM, inflammatory cell infiltration was reduced, and mast cell counts were decreased along with tissue edema compared with OVA mice. In the spleen, increased weight and inflammatory cells in the OVA mice were reduced in mice exposed to hHMSC and hHMSC-CM. No significant changes in villus/crypt length ratio of colon were seen.

It has been observed that Th2 immune response plays an important role in the pathogenesis of food allergy, similar to other allergic diseases. Inflammation in the gut drives the secretion of inflammatory cytokines such as IL-33 and IL-25, which channels the immune system towards Th2 response [30]. IgE, IL-4, IL-5, IL13, IL-23α, and IL-31 levels in our study are known Th2-related molecules [31]. In our study, the IgE level, and the transcription of IL-4, IL-5, and IL-23α was increased in the OVA mice, which suggests a role in inducing anaphylactic response, inflammation, and mast cell infiltration based on histological findings.

In our study, the number of mast cells substantially increased in the SI and mLN of OVA mice based on flow cytometry and histological findings. Previous studies [32,33] reported that elevated mMCP-1 levels in the serum, combined with IL-4 and IL-23α transcription in the SI of OVA mice in our study, suggest that mast cells may play a critical role in food allergy. MSCs secrete TGF-β1, which inhibits IL-4-induced FcεRI expression of mast cells [23,34], thereby reducing mast cell degranulation in the AD mouse model. This suppression appears to be related to PGE2 and COX-2 signaling [23]. Topical application of MSC-CM decreased mast cell recruitment, activity, and release of histamine in a mouse model of allergic conjunctivitis [22]. Further studies are needed to investigate the effects of MSC on mast cells in SI.

Treg cell is a key component of oral tolerance and plays a major role in immune responses to allergens in food allergy. Many studies suggested that allergen desensitization can induce a shift from Th2 towards Th1 response, inducing allergen-specific IgG4 blocking antibodies and regulatory cells. Allergen desensitization not only decreases the levels of IgE and IL-4 but also increases the number of IgG4, IL-10, and Treg cells [35]. Interestingly, Treg cells increased with OVA and decreased with hHMSC or hHMSC-CM in the ear, mLN, and SI, whereas IL-10 and TGF-β1 mRNA levels were increased in the skin and SI, respectively. It appears that the OVA-induced immune reaction differs depending on the organ. Increased levels of IL-10 mRNA in the skin and TGF-β1 in the SI after treatment with hHMSC or hHMSC-CM may result from compensatory mechanisms of decreased number of Treg cells, suggesting the need for a further study. It is known that oral tolerance is induced by initial sensitization of allergen in the gut [36], and allergen sensitization through the skin induces systemic allergic responses [37]. Our food allergy model involves OVA-sensitization through the skin first. Thus, intragastric OVA challenge after skin sensitization may accelerate induction of Treg cells, which are not functionally mature. Dendritic cells capture antigen in oral tolerance and induce differentiation of naïve T cells into Treg cells [38]. In our study, OVA appears to induce differentiation and proliferation of immature Treg cells because it did not increase transcription of IL-10 and TGF-β1. Recent studies have reported that reprogramming of Treg cells to Th2-cell-like phenotype occurs after oral allergic sensitization [7], which may explain the temporary increase in the number of immature Treg cells by OVA. Although we did not determine the protein levels of IL-10 and TGF-b1, exposure to hHMSC or hHMSC-CM appears to enhance the expression of immunosuppressive cytokine IL-10 or TGF-β1 differently depending on the organs, based on allergic scores, anaphylactic responses, and histologic specimens indicating alleviation of allergic symptoms and signs by hHMSCs or its CM in OVA mouse model.

In the food allergy mouse model, inflammation decreases the villus/crypt length ratio because of irregular villi and hyperplasia of crypts. Alleviation of inflammation reduces crypt hyperplasia, and thereby increases the villus/crypt length ratio [23]. In our study, no significant changes in villus/crypt length ratio were detected after administration of hHMSC or hHMSC-CM.

In our study, hHMSC-CM had a greater effect than hHMSCs in inhibiting Th2 cytokines and IgE expression, increasing IL-10 and TGF-β1, and decreasing the population of mast cells and Treg cells. The results suggested that the soluble factors secreted by MSCs play a major role in regulating immunologic function in the food allergy model. In the study of human umbilical cord-derived MSCs in the food allergy model, allergic symptoms and IL-4, TNF-α mRNA levels, inflammatory cells, and goblet cells in the colon were decreased by MSCs and MSC-CM [39]. Similar findings were presented in other allergic disease models. In the AD model, MSC-derived exosomes reduced clinical symptoms, along with the levels of IgE and eosinophils, IL-4, IL-23, and TNF-α [40]. Moreover, in a mouse model of allergic airway inflammation, both CM and extracellular vesicles from human BM-MSCs or mouse BM-MSCs decreased the severity of airway hyperreactivity and lung inflammation [41]. Exosomes, which include protein, lipid, and RNA, seems to play a key role in anti-allergic effects. Exosomes secreted by MSCs upregulated secretion of IL-10 and TGF-β1 from peripheral blood mononuclear cells of asthmatic patients, promoting proliferation of Tregs [42]. In other studies, exosomes from MSCs reduced symptoms of OVA-induced AAI and TGF-β levels in mice [43]. As epigenetic regulation such as DNA methylation [44], histone acetylation [45], and microRNAs [46] contribute to the pathophysiology of food allergy by affecting T cell differentiation toward Th2 cell type, and microRNAs in exosomes from MSCs promote the differentiation of Tregs in asthmatic patients [47], epigenetic changes could contribute the effects of exosomes. Further studies are required to discover the exact role of exosomes in food allergy.

Though not analyzed in our study, MSC administration by oral gavage in one study successfully altered the abundance and composition of gut microbiota, which is associated with gut allergies. MSCs significantly reversed the higher levels of OVA-induced IL-4 and TNF-α mRNA expressed in the OVA. Treatment with MSC-CM by oral gavage partially restored the gut microbiota and goblet cells as well as commensal bacteria in the gut [39].

There was a limitation in our study. MSCs used in this experiment were obtained from one young healthy donor. The constituent of MSC-derived exosome or extravesicle could be variable depending on the MSC tissue origin, donor’s age, and immune status [48,49]. To overcome the epigenetic change issue, the current study results should be proven by using hMSCs obtained from different donors. Eventually, it is necessary to reveal the key effective substance in MSC-derived exosome to overcome limitations resulting from individual differences and to develop a cell-based therapeutic agent which can be clinically applicable.

These results suggest that hHMSC therapy may be a promising target in preventing food allergy. MSCs showed significant clinical improvement without serious adverse events in clinical trials of MSCs administrated subcutaneously to AD patients [50] and intravenously in cases of chronic urticaria [51]. Further studies are needed to elucidate the key substances released from hHMSCs to induce immune tolerance.

## Figures and Tables

**Table 1:** Comparison of allergy scoring, diarrhea, anaphylactic responses, and body temperature.

	Allergy Scoring (Point ^†^)	Diarrhea (Point ^††^)	Anaphylactic Responses (Point ^†††^)	Body Temperature (°C)
Control	0.50 ± 0.84	0.00 ± 0.00	0.00 ± 0.00	36.90 ± 0.20
OVA	4.50 ± 0.50	1.80 ± 0.80	2.50 ± 0.50	33.90 ± 1.40
OVA + MSC	3.80 ± 0.90	1.70 ± 0.70	1.90 ± 0.70	34.40 ± 1.70
OVA + MSC-CM	3.10 ± 1.00 *	1.30 ± 0.80	1.60 ± 1.00 *	34.40 ± 1.30

Mean ± standard, ^†^ 7-point Likert scale (0–7), ^††^ 4-point Likert scale (0–3), ^†††^ 5 point Likert scale (0–4), * *p* < 0.05 versus OVA. MSC, mesenchymal stem cell; MSC CM, mesenchymal stem cell culture medium; OVA, Ovalbumin.

## Data Availability

The data presented in this study are available on request from the corresponding author.

## Supplementary Materials

The following supporting information can be downloaded at: https://www.mdpi.com/article/10.3390/biomedicines10020511/s1, Table S1: Gene information, Table S2: Comparison of ear skin thickness, mast cell counts of ear skin, small intestine, and spleen weight.
