# Supplementary material for: Preventive Effects of a Human Hematopoietic Mesenchymal Stem Cell (hHMSC) Therapy in Ovalbumin-Induced Food Allergy
Source: Biomedicines. 2022 Feb 21;10(2):511. doi: 10.3390/biomedicines10020511 (PMC8962321; doi:10.3390/biomedicines10020511)
Supplement: Supplementary file 1 [file biomedicines-10-00511-s001.zip › biomedicines-1546575-supplementary.pdf]

Supplementary table

Table S1. Gene information.

No.	Gene	Gene Symbol	Accession No. (species)	
1	*IL-4*	Il4	NM_021283.2 (*Mus musculus*)	
2	*IL-5*	Il5	NM_010558.1 (*Mus musculus*)	
3	*IL-13*	Il13	NM_008355.3 (*Mus musculus*)	
4	*IL-10*	Il10	NM_010548.2 (*Mus musculus*)	
5	*TGF- β1*	Tgfb1	NM_011577.2 (*Mus musculus*)	
6	*IL-23*	Il23a	NM_031252.2 (*Mus musculus*)	
7	*IL-31*	Il31	NM_029594.1 (*Mus musculus*)	
8	*IFN-γ*	Ifng	NM_008337.4 (*Mus musculus*)	
9	*IL-12*	Il12a	NM_008351.3 (*Mus musculus*)	
10	*Actb*	Actb	NM_007393.5 (*Mus musculus*)	Reference gene

Table S2. Comparison of ear skin thickness, mast cell counts of ear skin, small intestine and spleen weight.

	Control	OVA	OVA+MSC	OVA+MSC CM
Ear skin thickness (μM)	264.03±10.74	356.13±48.39	346.96±28.20	313.20±26.53*
Mast cell counts of ear skin (Cells/HPF)	3.17±1.94	9.00±3.81	6.00±5.34	5.33±2.61
Mast cell counts of small intestine (Cells/HPF)	1.30±1.20	6.00±1.28	3.90±1.90	3.5±2.30
Spleen weight (g)	0.26±0.03	0.39±0.05	0.31±0.04	0.33±0.04

Mean±SD. * : P < 0.05 versus G2. Cells/HPF, mast cells per high power field (x400)
MSC, mesenchymal stem cell; MSC CM, mesenchymal stem cell culture medium; OVA, Ovalbumin
